# *Smcr8* deficiency disrupts axonal transport-dependent lysosomal function and promotes axonal swellings and gain of toxicity in C9ALS/FTD mouse models

**DOI:** 10.1093/hmg/ddaa012

**Published:** 2020-02-14

**Authors:** Chen Liang, Qiang Shao, Wei Zhang, Mei Yang, Qing Chang, Rong Chen, Jian-Fu Chen

**Affiliations:** 1 Center for Craniofacial Molecular Biology, University of Southern California (USC), Los Angeles, CA 90033, USA; 2 Department of Cellular Biology, University of Georgia, Athens, GA 30602, USA; 3 Department of Diagnostic Radiology and Nuclear Medicine, University of Maryland School of Medicine, 100 N. Greene Street, Baltimore, MD 21205, USA

Since article was originally published online, some minor corrections which had been missed at the proof stage (due to OUP error) have since been updated: 1) In Fig, 5F, WT, and Smcr8-/- have been added into the labels, and also *, n.s. in the notes; 2) In Fig. 9C, GR has been amended to GP following a re-calculation, because the authors found that previous GR antibody recognizes non-specific autofluorescent proteins. The modifications did not change the conclusions and interpretation of the results.

